# Supplementary material for: Comprehensive Annotation and Functional Exploration of MicroRNAs in Lettuce
Source: Front Plant Sci. 2021 Dec 24;12:781836. doi: 10.3389/fpls.2021.781836 (PMC8739914; doi:10.3389/fpls.2021.781836)
Supplement: Supplementary Data 1 — Results of categories 0–2 from PARE-Seq analysis (including three subfiles:1_1, 1_2, 1_3). [file Data_Sheet_10.ZIP › GSM2230756.plot/Lsa-miRN33_Lsat_1_v5_gn_9_120700.1_1108_TPlot.pdf]

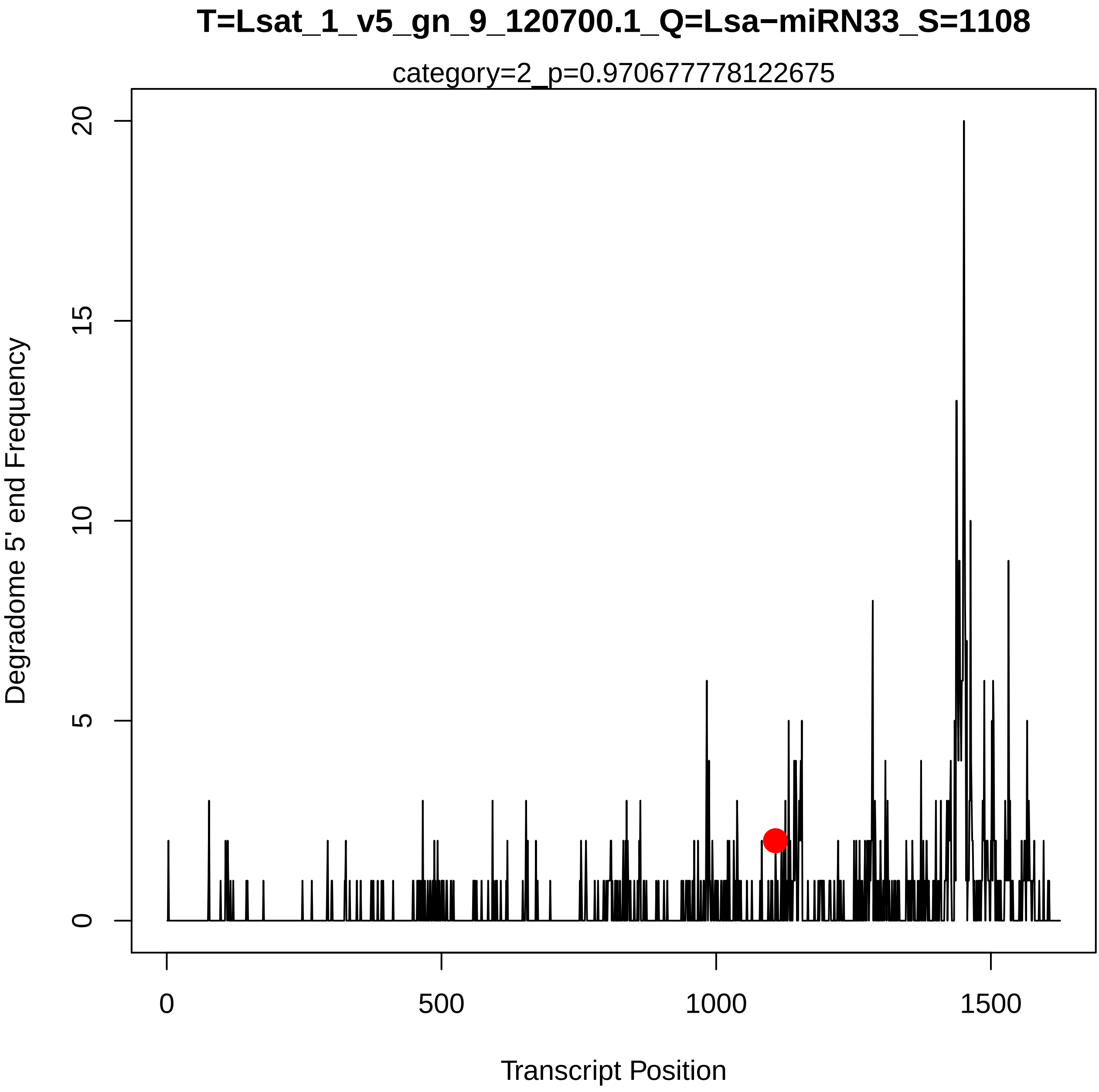

T=Lsat_1_v5_gn_9_120700.1_Q=Lsa−miRN33_S=1108
category=2_p=0.970677778122675
Degradome 5' end Frequency
0
5
10
15
20
0
500
1000
1500
Transcript Position
